# 

**Published:** 1917-10

**Authors:** 


### TABLE OF CONTENTS

Tuberculosis, A Disease of Children ---------------------191

Tuberculosis, A Disease of Children ---------------------191

Constructive Suggestions Toward The Control of Syphilis, Gonorrhea, Pneumonia, Malaria, Typhoid, and Typhus Fever ---------------------200

Some Observations on Salpingitis ---------------------209

Fracture Don'ts ---------------------215

Medical Items ---------------------222

